# Optical Microscopy for High-Resolution IPMC Displacement Measurement

**DOI:** 10.3390/s26020436

**Published:** 2026-01-09

**Authors:** Dimitrios Minas, Kyriakos Tsiakmakis, Argyrios T. Hatzopoulos, Konstantinos A. Tsintotas, Vasileios Vassios, Maria S. Papadopoulou

**Affiliations:** 1 Department of Information and Electronic Engineering, International Hellenic University (IHU), 57400 Thessaloniki, Greece; dimitrovitsm@gmail.com (D.M.); ahatz@ihu.gr (A.T.H.); vassios@ihu.gr (V.V.); mspapa@ihu.gr (M.S.P.); 2 ELEDIA@AUTH, School of Physics, Aristotle University of Thessaloniki, 54124 Thessaloniki, Greece

**Keywords:** electronic circuit, micro-displacement measurement, tip tracking algorithm, optical displacement sensor

## Abstract

This study presents an integrated, low-cost system for measuring extremely small displacements in Ionic Polymer–Metal Composite (IPMC) actuators operating in aqueous environments. A custom optical setup was developed, combining a glass tank, a tubular microscope with a 10× achromatic objective, a digital USB camera and uniform LED backlighting, enabling side-view imaging of the actuator with high contrast. The microscopy system achieves a spatial sampling of 0.536 μm/pixel on the horizontal axis and 0.518 μm/pixel on the vertical axis, while lens distortion is limited to a maximum edge deviation of +0.015 μm/pixel (≈+2.8%), ensuring consistent geometric magnification across the field of view. On the image-processing side, a predictive grid-based tracking algorithm is introduced to localize the free tip of the IPMC. The method combines edge detection, Harris corners and a constant-length geometric constraint with an adaptive search over selected grid cells. On 1920 × 1080-pixel frames, the proposed algorithm achieves a mean processing time of about 10 ms per frame and a frame-level detection accuracy of approximately 99% (98.3–99.4% depending on the allowed search radius) for actuation frequencies below 2 Hz, enabling real-time monitoring at 30 fps. In parallel, dedicated electronic circuitry for supply and load monitoring provides overvoltage, undervoltage, open-circuit and short-circuit detection in 100 injected fault events, all faults were detected and no spurious triggers over 3 h of nominal operation. The proposed microscopy and tracking framework offer a compact, reproducible and high-resolution alternative to laser-based or Digital Image Correlation techniques for IPMC displacement characterization and can be extended to other micro-displacement sensing applications in submerged or challenging environments.

## 1. Introduction

Ionic Polymer-Metal Composites (IPMCs) are a class of soft electroactive polymers which deform under low-voltage electrical excitation due to the movement of hydrated cations within their ionic matrix [1,2]. Their distinctive bending behavior, often compared with biological muscle contractions, results from their unique swelling response due to mobile ions and surface electrodes [3,4]. Owing to their low power consumption, high flexibility and compatibility with wet environments, IPMCs have been widely investigated for applications in soft robotics, biomedical devices, biomimetic actuators and underwater sensing systems [5,6,7,8,9].

Understanding and optimizing their actuation behavior, however, requires not only precise and reliable displacement measurement techniques but also high-resolution camera systems and well-designed actuation control electronics, particularly when operating in submerged or dynamic environments [10,11].

Laser-based approaches, most notably Laser Doppler Vibrometry (LDV), remain among the most precise tools for displacement metrology in smart materials. Modern LDV instruments routinely achieve sub-micron resolution while capturing high-frequency vibrations in real time, which explains their broad use in characterizing the response of IPMC actuators to changing stimuli or environments [12,13]. The principle is succinct: a Doppler shift in the back-scattered beam is converted to velocity and then integrated to displacement, so motion is monitored without physical contact and with very high fidelity.

Even so, immersion complicates optics. Refractive-index discontinuities at interfaces, scattering from particulates or microbubbles, and tight alignment tolerances can depress accuracy markedly [14]. In parallel, complete LDV setups tend to be expensive, comparatively large, and prescriptive about environmental stability; compact or low-budget benches cannot always accommodate the footprint and upkeep, which limits adoption in small experimental rigs [15].

Beyond laser instrumentation, capacitive displacement sensors offer a mature pathway to high precision. These devices infer motion from fluctuations in capacitance as the gap between conductive elements varies, enabling sub-micron sensitivity when grounding, shielding, and thermal conditions are well regulated [16]. Their small form factor, quick response, and moderate price have encouraged us to use them across smart-material and micro-actuator studies [17].

However, humid or submerged settings introduce leakage and electrical interference that erode signal stability [18]. Because the transducer is contact- or near-contact by geometry, it can also perturb the natural bending of soft ionomer laminates, which undermines fidelity for very small underwater motions [19]. In electrically noisy tanks, repeatability at the micrometer scale may be compromised despite careful shielding or encapsulation.

To sidestep such electrical pathways, fiber-optic displacement sensors encode motion as changes in optical intensity, phase, or interference fringes guided through fibers [20,21]. Owing to strong isolation from electromagnetic interference and compact packaging, they have gained traction in biomedical engineering, robotics, and industrial diagnostics, where moisture and noise are routine rather than exception [22,23,24]. Yet trade-offs persist: alignment is delicate, interrogators and couplers raise cost, and mechanical disturbance or thermal drift can bias calibration during long experiments. Still, when submersion or EMI renders conventional probes impractical, fiber optics are often the sensible choice.

Ultrasonic and acoustic techniques support displacement estimation where line-of-sight is limited. By analyzing reflections or transmissions to recover time-of-flight, phase changes, or frequency modulations, they infer motion even in opaque media [25,26]. Underwater operation is well suited to these methods because acoustic waves are largely insensitive to optical distortions, enabling precise timing analysis. Nevertheless, their native spatial resolution is typically coarser than optical methods, which makes detection of micrometer-scale IPMC deflections challenging; in practice, they serve as complementary rather than primary instruments for the finest motions.

Magnetic-field solutions, including Hall-effect sensors and planar magnetic grids, have likewise been engineered for displacement over multiple degrees of freedom or broad ranges [27,28,29,30]. They are robust, alignment-tolerant, and easy to integrate in mechatronic assemblies. The penalty is resolution: noise floors and calibration drift usually preclude reliable measurement of micro-deformations characteristic of IPMC actuation, confining their role to coarse kinematics or structural calibration tasks.

DIC functions by tracking surface-pattern deformation between successive images to extract strain and displacement fields with high spatial richness. In that sense, Digital Image Correlation provides a full-field alternative to point sensors and suits complex geometries and large-area deformations across scales and materials [31,32,33]. Yet the underwater case raises the bar: durable high-contrast speckles, uniform illumination, and high-resolution cameras are typically required; refraction at fluid/air boundaries must be modeled or corrected, otherwise accuracy falls. Consequently, integrating DIC for submerged IPMCs demands additional calibration and environmental compensation that not every lab can readily support.

Taken together, these options outline familiar trade-offs among precision, complexity, and environmental tolerance. LDV offers submicron precision but is vulnerable to refraction, scattering, and alignment issues in water on top of cost and footprint [12,13,14,15]. Capacitive sensors excel in dry, controlled conditions yet become inaccurate under humidity or submersion due to electrical interference and specimen coupling [16,17,18]. Fiber-optic methods yield excellent isolation but require nontrivial alignment and specialized interrogators, which can render laboratory studies cost-prohibitive for smaller teams [20,21,22,23]. Ultrasound and acoustics work reliably in water but lack the spatial resolution needed for the smallest IPMC displacements [25,26]. DIC provides powerful full-field data, though with imaging and calibration overheads that complicate compact experiments [31,32,33].

Against that backdrop, an economical, reproducible, high-resolution approach tailored to underwater IPMC characterization is desirable. Accordingly, an optical pathway was developed that emphasizes simplicity alongside precision and scalability. The arrangement employs a tubular microscope with a moderate-magnification achromatic lens and a digital USB camera, together with uniform LED backlighting to render a sharp side-view silhouette for displacement capture. With straightforward calibration, edge-based detection supports high-resolution tracking of the free-end trajectory under realistic drive signals.

In contrast to LDV or fiber-interferometric instruments that demand intricate calibration and specialized apparatus, the proposed method targets comparable accuracy while remaining cost-effective and operationally consistent. Unlike capacitive and ultrasonic techniques, the system sidesteps electrical interference problems and ensures consistent performance in underwater conditions. Moreover, unlike Digital Image Correlation, our approach does not rely on expensive high-speed cameras or complex surface patterning, making it suitable for laboratory-scale testing and prototype development.

Despite the availability of these methods, there is still a lack of a dedicated, low-cost optical microscopy system specifically optimized for underwater IPMC displacement characterization that combines sub-micrometer spatial resolution, real-time tracking capability and integrated electronic protection. Existing vision-based approaches either target dry or quasi-static conditions, or rely on expensive, laboratory-grade optics and cameras that are not easily reproducible in compact experimental settings [10,11,31,32].

Table 1 presents a concise comparison of representative techniques used for IPMC and related ionic polymer actuator displacement/strain measurements, reporting indicative accuracy/spatial resolution, qualitative cost, and key pros/cons. This overview positions our optical microscopy-based tip tracking approach, which targets sub-micrometer spatial resolution with a relatively low-cost setup and real-time tracking capability for underwater applications.

The main contributions of this work are as follows: (i) the design and implementation of a custom tubular microscopy setup with uniform LED backlighting, achieving spatial sampling on the order of 0.5 μm/pixel for side-view IPMC displacement measurement in water; (ii) the development of a predictive, grid-based tip tracking algorithm that exploits a constant-length geometric constraint to provide robust, real-time localization of the IPMC free end; (iii) the integration of a dedicated IPMC driver and monitoring circuit with overvoltage, undervoltage, open-circuit and short-circuit detection for safe long-term operation; and (iv) a comprehensive experimental evaluation of the system, including spatial resolution, lens distortion, processing time and detection accuracy under realistic actuation conditions.

The remainder of this paper is organized as follows. Section 2 describes the optical microscopy setup, the IPMC driver and fault-protection circuitry, and the proposed tip tracking algorithm. Section 3 presents the experimental results obtained for spatial resolution, real-time tracking performance and fault detection. Section 4 summarizes the main conclusions and outlines directions for future work.

## 2. Materials and Methods

### 2.1. Experimental Setup of Microscopy System

**Figure 1:**
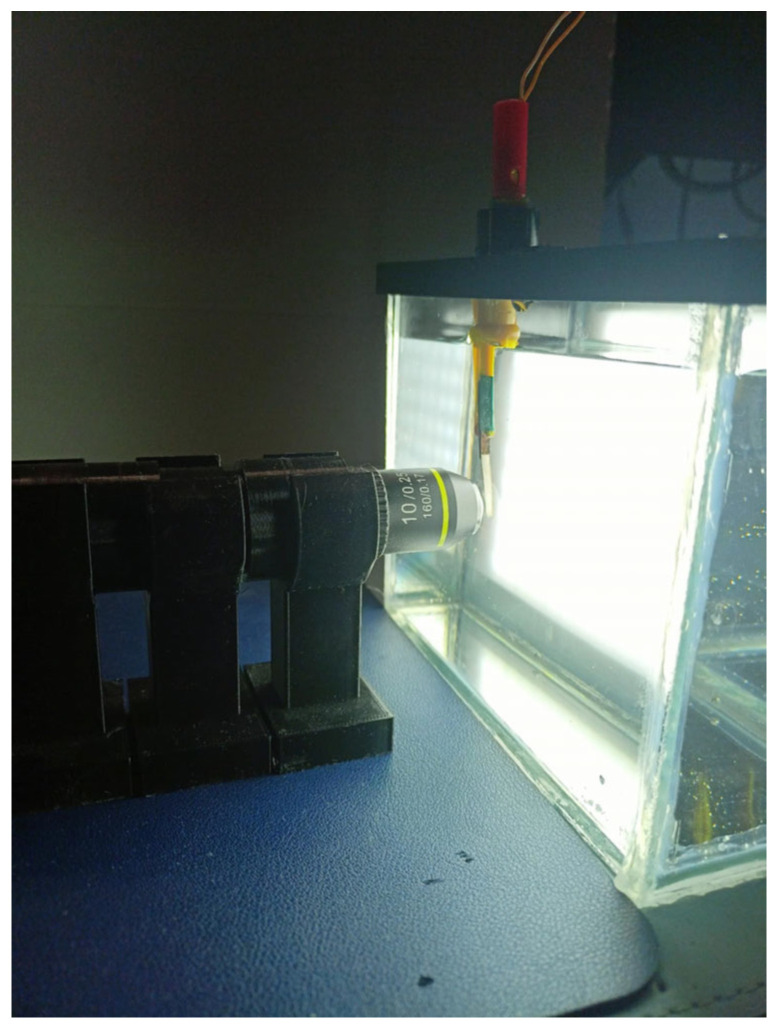
Experimental Setup.

As illustrated in Figure 1, a custom optical setup was developed to measure the displacement of an Ionic Polymer-Metal Composite (IPMC) actuator operating in liquid environments. To simulate realistic working conditions while decreasing electrical conductivity and optical interference, this actuator was submerged in a transparent glass tank filled with deionized water for immersion, with dimensions of 12 cm height, 7 cm width, and 15 cm in length (external dimensions of this tank).

IPMC was secured using a custom-made clip designed specifically for underwater mounting and reliable electrical contact, featuring built-in copper electrodes which acted as terminals for its electrical excitation. Furthermore, an actuator was positioned so its side view was visible through a microscope for direct measurement and observation of lateral displacement.

**Figure 2:**
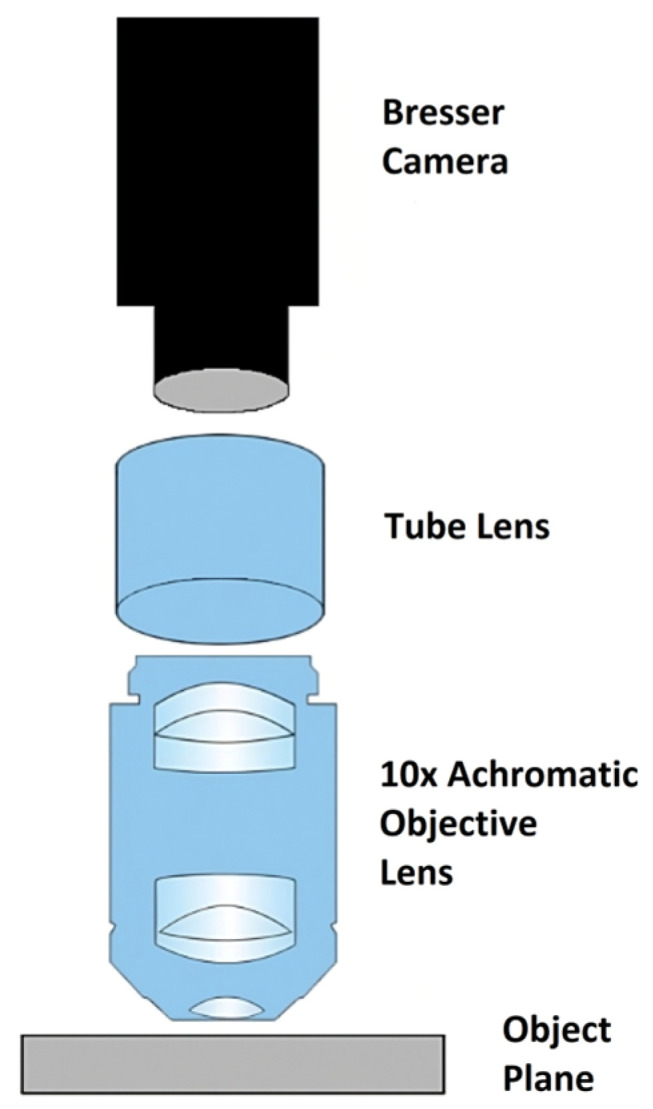
The custom-built tubular microscope.

For imaging, the imaging system employed a specially designed tubular microscope with an overall tube length of around 84 mm. It was chosen due to its compact design and ease of alignment along a single optical axis. Equipped with a 10× achromatic objective lens which provided magnification while simultaneously minimizing chromatic and spherical aberrations and keeping distance between objective and IPMC sample within an ideal 7.5–10 mm range due to thickness of glass wall (4 mm) and lens’ ideal focusing range (Figure 2).

A Bresser digital camera was integrated directly into the microscope tube at its upper end. This camera model was selected because it is specifically tailored to seamlessly integrate into tubular microscopes without requiring an eyepiece, providing real-time digital capture of magnified images.

To maintain an undistorted optical path, the microscope was mounted on the side wall of the tank at an angle that maintained a 1 mm gap from its outer surface to minimize distortion and ensure uniform focus across sample plane.

A custom-designed backlight panel was installed on the opposite tank wall and aligned directly behind the sample relative to the camera, covering its full external area (7 cm × 12 cm) consisting of a 15 × 9 grid of true white LEDs. This setup was essential for proper microscope operation as this type of objective lens needs transmitted light (illumination from behind an object) in order to render images visible. Without backlighting, images captured through viewing paths would appear nearly black due to inadequate light transmission.

All measurements were performed under constant illumination using the dedicated backlight panel. Prior to each experiment, illumination conditions were verified through a short screening step to ensure that the IPMC contour and the endpoints of interest were clearly distinguishable. If the contrast/visibility criteria were not met, the experiment was not conducted, as reliable edge and tip detection would be compromised.

Spatial calibration was performed by placing a calibrated micrometer slide directly inside the glass tank filled with water, at the same position and depth as the IPMC sample. Consequently, refraction effects due to the glass wall and the water medium, as well as the glass thickness, are inherently included in the pixel-to-length calibration factor. The reported spatial resolution therefore already accounts for these optical effects, and no additional refraction correction is required.

The glass tank was placed on a stable surface to ensure mechanical stability during measurements. After installation of the IPMC clip and filling of the tank, measurements were performed only after transient water motion had completely subsided—particularly during spatial calibration and parameter extraction prior to initiating IPMC actuation—in order to avoid disturbances caused by water waves or handling. Additionally, the proposed tip-detection algorithm remains capable of reliably tracking the IPMC free end in the presence of minor disturbances during the experiments, even while the actuator is moving.

### 2.2. IPMC Driver and Measurement Circuit

**Figure 3:**
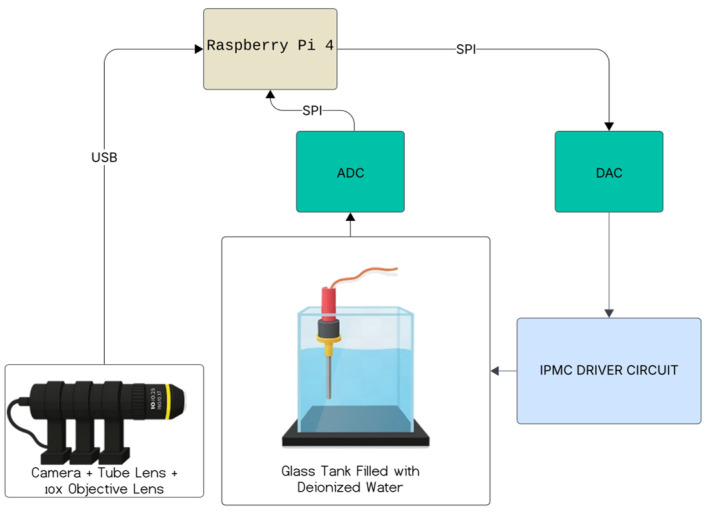
The block diagram of the measurement system.

Figure 3 illustrates the block diagram showing the setup of the measurement system designed for simultaneous excitation and monitoring of an IPMC actuator. The signal pathway begins at the Raspberry Pi computer serving as the central control unit. Through the SPI interface the Raspberry Pi sends data to a Digital-to-Analog Converter (DAC) which converts the input into an analog excitation signal. The analog signal produced by the DAC is then directed to a push/pull amplifier for supplying the required current to the IPMC specimen. The left terminal of the IPMC connects to the output of the amplifier whereas the right terminal passes through a 1 Ω shunt resistor, which is used to measure the current, in operation. The voltage drop across the shunt is sent to an Analog-to-Digital Converter (ADC) which converts the analog voltage into a signal for transmission back to the Raspberry Pi, for data logging and evaluation. At the time a high-definition digital camera captures the mechanical movement of the IPMC viewing the specimen through the wall of the glass tank. The optical setup includes a microscope fitted with a 10× objective lens and an LED backlight system delivering a sharp image of the sample for later image analysis and displacement measurement. The camera is linked to the Raspberry Pi, allowing for the simultaneous collection of both visual and electrical information.

The diagram illustrates the interconnectivity and collaboration of all subsystems within a cohesive framework, enabling thorough tracking and evaluation of the electro-mechanical characteristics of the IPMC. Alongside signal generation and data collection, the framework incorporates specific monitoring and safeguarding routes to identify and address unusual electrical conditions throughout extended experiments.

**Figure 4:**
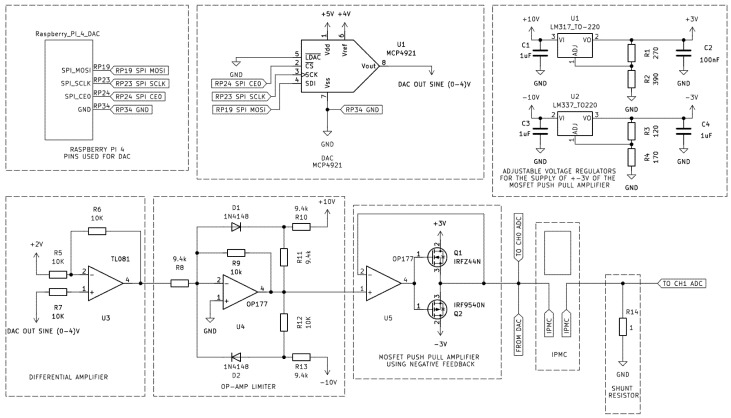
IPMC Control Circuit.

To drive the IPMC actuator and record at the same time its electrical behavior, a custom signal generation and measurement circuit was developed, as shown in Figure 4. A Raspberry Pi 4 Model B (8 GB RAM) was used as the main control unit. It is utilized via SPI with an MCP4921 12-bit Digital-to-Analog Converter (DAC), which was configured to output a sinewave ranging from 0 to 4 V. The DAC’s reference voltage (VREF) was provided by a low-noise buffer circuit, ensuring signal stability.

The single-ended sinewave produced by the DAC was first transformed into a bipolar signal centered around 0 V through a differential amplifier, having as a result a waveform in the range of −2 V to +2 V. This signal was then routed to a push-pull amplifier stage consisting of complementary MOSFETs (IRFZ44N for the positive half-cycle and IRF9540N for the negative), powered by a ±3 V supply. To safeguard the IPMC against overvoltage conditions, the driver control circuit incorporates an op-amp-based precision limiter stage. This circuit employs an OP177 operational amplifier in combination with two 1N4148 diodes and a resistor feedback network. Unlike simple Zener limiter configurations, this setup ensures more accurate and symmetrical voltage clamping around the desired threshold, while minimizing distortion in the linear operating region. The limiter effectively restricts the amplitude of the excitation signal before it reaches the push/pull power amplification stage, thereby protecting both the IPMC and the rest circuitry from potentially damaging voltage excursions. The ±3 V supply rails were derived from the ±10 V power source through adjustable voltage regulators (LM317 and LM337) in order maintain stable and safe voltage output levels even in the event of limiter failure.

**Figure 5:**
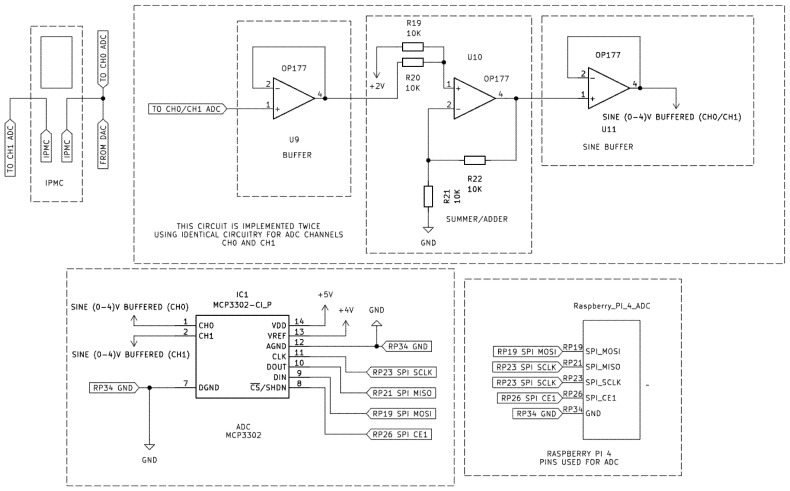
ADC control circuit used for signal conditioning. Identical circuitry is employed for ADC channels CH0 and CH1.

The output of the push-pull amplifier was directly connected to one terminal of the IPMC, while the opposite terminal was connected to ground via a 1 Ω, 5 W shunt resistor, enabling real-time current sensing. Simultaneously, both IPMC terminals were connected to separate input channels (CH0 and CH1) of an MCP3302 13-bit ADC to facilitate differential voltage measurement. Before reaching the ADC, each signal was processed through an identical signal-conditioning circuit consisting of a buffer stage, followed by a summing amplifier that shifted the bipolar ±2 V waveform to a unipolar 0–4 V range. Before being sampled, the conditioned signal passed through a final buffer stage to ensure impedance matching and signal integrity at the ADC input. The ADC then communicated back to Raspberry Pi for synchronized data acquisition. The related signal-conditioning and ADC-interfacing schematics for channels CH0 and CH1 are depicted in Figure 5.

This op-amp stage shifted the waveform to the 0–4 V range, so its input is ideal for the MCP3302 13-bit Analog-to-Digital Converter (ADC), which only works with positive voltages.

The operational amplifier stages were powered with ±10 V dual supply, while the digital components (MCP4921 DAC and MCP3302 ADC) were powered at +5 V, regulated via a 7805-voltage regulator. This ensured consistent voltage levels across all digital logic and analog interfaces.

To minimize the impact of electrical noise, we used stable backlight illumination and applied standard grounding and decoupling practices in the driver circuitry. Any residual electrical noise, even if present, is mainly concentrated at higher frequencies and therefore results in only very small and practically negligible displacement compared to the controlled, low-frequency actuation examined in this work.

### 2.3. Fault Detection in the IPMC Control Circuit

Reliable long-term displacement measurements in aqueous media require not only precise optical tracking, but also robust protection of the IPMC sample and the associated driver electronics. Abnormal operating conditions such as supply overvoltage or undervoltage, amplifier saturation, and poor contact of the clip electrodes can lead to erroneous displacement data or even permanent damage to the actuator. For this reason, the IPMC control circuit incorporates a dedicated fault detection subsystem that continuously monitors the supply rails, the amplifier output and the current load. The following subsections describe the four main fault modes considered and the corresponding detection mechanisms implemented in hardware and firmware.

#### 2.3.1. Fault Detection of the 5 V Supply from the LM7805

**Figure 6:**
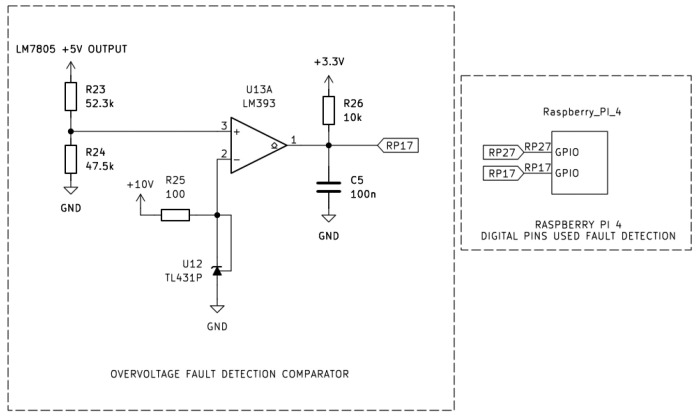
Overvoltage fault detection comparator using the LM393 to validate the LM7805 over 5 V output range.

**Figure 7:**
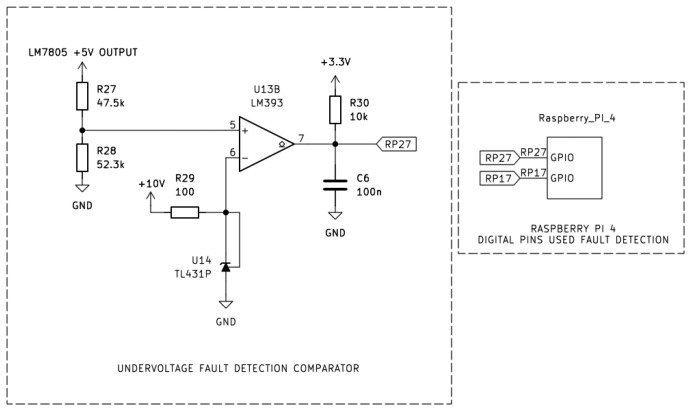
Undervoltage fault detection comparator using the LM393 to validate the LM7805 under 5 V output range.

As shown in Figure 6 and Figure 7, the design employs two comparator stages based on the LM393 dual differential comparator, and threshold stability is set by a TL431 precision shunt reference of 2.495 V. In both configurations, the non-inverting input (+) of each comparator was connected to the 5 V rail generated by the LM7805 voltage regulator, while the inverting input (−) was connected to the TL431 reference output.

Figure 6 shows the overvoltage detection circuit, the 5 V rail coming from the LM7805 is sensed via a 52.5 kΩ/47.5 kΩ divider. When the output of the LM7805 exceeds the 5.25 V threshold, the divider output rises above the 2.495 V reference voltage and as a result the LM393 output transistor turns off and the open-collector output is being “pulled up” to 3.3 V ensuring a logical HIGH to the Raspberry Pi (RP17), thereby flagging an overvoltage condition.

Figure 7 shows the undervoltage detection circuit, the 5 V rail coming from the LM7805 is sensed via a 47.5 kΩ and 52.3 kΩ divider. When the output of the LM7805 drops below 4.75 V threshold, divider output falls below the 2.495 V reference, having as a result the LM393 output transistor to conduct. Consequently, the output is being “pulled low”, generating a logical LOW signal that was sent to the pin RP27 digital input pin of the Raspberry Pi 4. Both comparator outputs employed 3.3 V pull-up resistors to ensure compatibility with the Raspberry Pi’s GPIO logic levels.

#### 2.3.2. ADC Fault Detection on the Push/Pull Output

A second fault detection mechanism was implemented using the CH0 input of the MCP3302 Analog-to-Digital Converter (ADC), in order to continuously monitor and validate the output of the push–pull amplifier stage. Since the amplifier operates with a ±3 V supply and is driven by a ±2 V sinusoidal waveform, the expected output voltage remains between the nominal range of operation. Any variation exceeding these voltage limits is automatically considered an indicator of malfunction, whether due to amplifier malfunction, load conditions beyond acceptable parameters or short circuit behavior at IPMC terminals. This monitoring strategy enables real-time verification of operational integrity through direct feedback to the control system.

#### 2.3.3. Clip Copper Contacts Not Touching (Open Circuit)

A third fault detection method was implemented to identify open-circuit conditions at the IPMC terminals. The detection is based on monitoring the voltage across the 1 Ω shunt resistor (R14), as depicted in Figure 5, through the CH1 input of the MCP3302 13 bit ADC. Under normal operating conditions, the shunt resistor exhibits a time-varying voltage corresponding to the sinusoidal current drawn by the IPMC. However, if the clip contacts are open and the circuit is no longer continuous, and the measured voltage remains at 0 V.

Since the driving signal is a ±2 V sinusoidal waveform, which naturally crosses zero during each period, a false fault indication could occur if the measurement is taken during the zero-crossing interval. To avoid such misclassification, a time window of 700 ms corresponding to half the period of the excitation waveform at 0.7 Hz ($T=\frac{1}{0.7}=1.43\;s$) is introduced. If no measurable voltage variation is detected after this interval, an open-circuit fault flag is raised on the control interface, indicating disconnection or improper contact of the IPMC sample.

#### 2.3.4. Clip Copper Contacts Touching (Short-Circuit)

A fourth fault detection mechanism was incorporated to identify short-circuit conditions occurring when the clip contacts touch each other in the absence of the IPMC sample. In this scenario, the entire ±2 V sinusoidal waveform is applied directly across the 1 Ω shunt resistor (R14), as illustrated in Figure 5 and monitored via the CH1 input of the MCP3302 ADC. This condition results in an excessive current of approximately 2 A, indicating a direct short between the amplifier output terminals.

To prevent damage to the circuit, the ADC continuously monitors the shunt voltage and compares it with the expected current range. When a current exceeding the preconfigured threshold is detected, a short-circuit fault is being flagged by the control system. As an additional protective measure, the DAC responsible for generating the 0–4 V sinusoidal excitation waveform is immediately disabled, effectively stopping the signal delivery to the amplifier and isolating the fault condition and preventing further potential damage.

All four fault detection mechanisms are incorporated into a unified fault monitoring interface, providing the user with real-time visual feedback on the system’s functioning condition. Through this interface, active fault conditions are clearly indicated, allowing immediate identification of overvoltage, undervoltage, open-circuit, or short-circuit events. Once a fault is resolved, the user is given the option to enable the DAC again directly from the control panel, thereby restoring normal operation without requiring a full system restart. This integrated approach ensures both operational safety and user convenience, while maintaining continuous supervision of the experimental setup.

### 2.4. Predictive Localization of IPMC Tip Displacement Using a Grid-Based Adaptive Search Strategy

**Figure 8:**
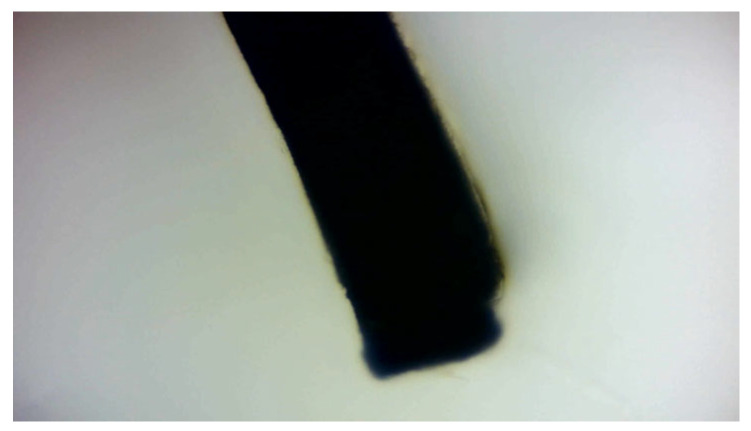
Representative capture of the Nafion-based, platinum-plated IPMC sample through the microscopic imaging system, where the actuator geometry, the anchoring base and the free lower end are clearly distinguished.

Figure 8 shows a representative frame of the IPMC sample as recorded by the high-resolution microscopy system. The imaging setup enables precise observation of the actuator’s shape, position and bending, providing the necessary basis for edge-detection techniques to identify the free lower tip through image processing.

**Figure 9:**
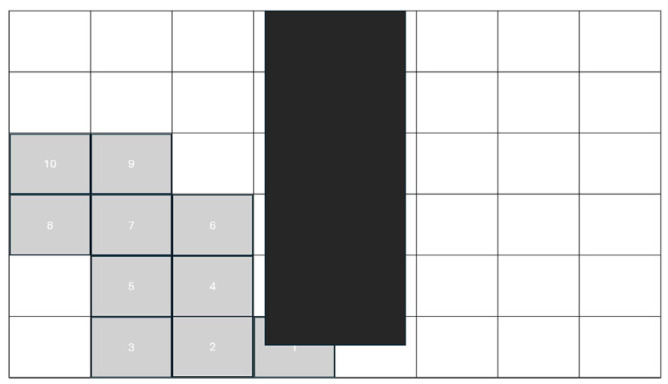
Indicative illustration of the grid-based prediction and edge-detection algorithm for the IPMC tip. The image shows the division of the region of interest into a grid, the estimated position of the tip, as well as the candidate search cells defined by the algorithm for the next frame.

Figure 9 schematically illustrates the operation of the detection algorithm: the region of interest is divided into a fixed-size grid and the cells that are most likely to contain the IPMC tip are highlighted. This estimation relies on the previous tip position, the geometric constraint imposed by the actuator length and the expected bending trajectory, enabling fast and reliable localization of the point of interest even in the presence of abrupt motion changes. In the following, this method is referred to as a grid-based tip tracking algorithm.

Each frame (1920 × 1080, 8-bit grayscale) is first normalized by a robust linear contrast stretch (1st–99th percentile) to compensate for slow illumination drift and minor LED intensity fluctuations. A light Gaussian smoothing (σ ≈ 1.0–1.2 pixels) is then applied to suppress sensor noise while preserving high-gradient edges along the IPMC contour.

A binary mask of the actuator is obtained using adaptive thresholding (local window ≈ 25–35 pixels), followed by morphological opening and closing with a 3 × 3 structuring element. These operations remove isolated speckles and fill sub-pixel gaps along the contour, yielding a clean, single-blob representation of the IPMC in each frame.

From this mask, a refined contour is extracted using Canny edge detection restricted to the foreground region. To estimate the distal end, the mask is first skeletonized and the skeleton is parameterized along its arc length. Only the last 10–15% of the skeleton, corresponding to the free lower part of the actuator, is retained, and Harris corner responses are computed along the associated contour segment. Candidate tip points are defined as corners within this distal band. Among these candidates, the tip is selected as the point that maximizes a joint score combining (i) Harris “cornerness” and (ii) distance along the centerline from the fixed base, thus favoring strong corners located near the free end of the IPMC.

Let A denote the fixed base point of the actuator and L the IPMC length measured in the first frame, which is assumed constant over time (inextensibility constraint). The base point A is defined by the mechanically clamped anchoring region and remains stable in our setup. For completeness, any small residual drift can be compensated by re-detecting A in each frame from the proximal skeleton endpoint within a small ROI around the clamp (anchoring region) and computing the tip displacement relative to this reference. The tip position at discrete time step *t* is denoted by p*_t_*, while p*_t_*_−1_ is the position in the previous frame. The instantaneous displacement vector is given by Equation (1).

(1) $$d_{t}=p_{t}-p_{t-1}$$

An unconstrained prediction of the next tip position is then obtained by linear extrapolation of the recent motion, as expressed in Equation (2).

(2) $${\tilde{p}}_{t+1}=p_{t}+d_{t}$$

However, the IPMC is assumed to bend without changing its length, so the tip must always lie on the geometric locus of points at distance *L* from the base A. This constraint defines a circle, given in Equation (3).

(3) $$C=\{x\in R^{2}:∥x-A∥=L\}$$

The constant-length constraint is employed only to initialize and bound the tip search between consecutive frames (prediction prior). The final tip localization remains feature-driven, and when the prediction is insufficient the adaptive grid search is automatically expanded, so small apparent length variations do not bias the measured displacement.

The final predicted position is obtained by projecting the unconstrained estimate onto this circle, as shown in Equation (4). This projection enforces inextensibility and regularizes the prediction when the motion is noisy or temporarily corrupted.

(4) $${\hat{p}}_{t+1}=A+L\;\frac{{\tilde{p}}_{t+1}-A}{∥{\tilde{p}}_{t+1}-A∥}$$

To reduce the computational cost of tip detection, the image is partitioned into a fixed grid of rectangular cells (e.g., 20 × 20 pixels). Before processing the video sequence, all cells that intersect the circle are pre-indexed; only these cells can contain a physically valid tip position. In addition, a short trajectory buffer of the last *K* tip positions (typically *K* ≈ 5–8) is maintained, approximating the recent path of the actuator.

At each time step, the cell containing the predicted tip is identified and assigned highest priority. The remaining candidate cells are then ranked according to three criteria: (i) geometric feasibility, giving priority to cells intersecting the circle; (ii) proximity to the prediction, favoring cells whose centers are closest to the predicted position; and (iii) consistency with the past trajectory, promoting cells that have recently contained the tip or are 4-connected neighbors of such cells.

The tip search starts from the highest-priority cell. Within each visited cell, all contour points belonging to the IPMC mask are re-examined locally: Canny edge detection and Harris corner computation are restricted to the cell window, and the same joint scoring (cornerness and distal distance along the skeleton) is used to identify the best tip candidate. If a valid tip is found in the first cell, the search terminates immediately, minimizing computation.

If no valid tip is detected in the predicted cell, the search area is expanded adaptively by examining successive “rings” of neighboring cells around the prediction. At expansion level *r*, all cells that are *r* steps away in 4-connected grid distance are visited, but only among those that intersect the circle. This process continues until either (i) a new tip is found or (ii) a maximum expansion level is reached (in practice, ≤3–5 rings under nominal motion). Because the expansion is guided simultaneously by the geometric constraint and the recent trajectory, the algorithm remains robust even under abrupt motion changes or partial occlusion of the tip.

Once the tip is localized, the new position p_t+1_ is stored in the trajectory buffer and used to update the displacement vector and the next prediction. The entire procedure—preprocessing, candidate extraction within grid cells, constrained prediction and adaptive search—is repeated for every frame of the sequence. In this way, the algorithm yields a smooth, time-ordered sequence of tip positions that respects the constant-length constraint and can be directly converted into displacement time series for further analysis in Section 3.

## 3. Results

The camera recorded video at 1920 × 1080 pixels and 30 frames per second (fps). A micrometer calibrated slide with 10 µm divisions (0.01 mm) was used to determine the system’s field of view. For an image size of 1920 × 1080 pixels, the measured field of view was approximately 1.03 mm (width) × 0.56 mm (height), from which the spatial resolution along each axis was calculated. The corresponding parameters and results are summarized in Table 2.

A total of 100 independent measurements of spatial resolution (µm/pixel) were conducted, from which the mean values and confidence intervals were derived. The final value for spatial resolution on the horizontal axis was determined to be 0.536 ± 0.001 µm/pixel (95% confidence interval) reflecting high stability and accuracy during measurement in the central region of the image. Lens distortion was limited with maximum edge deviation of +0.015 μm/pixel (≈+2.8% relative to the center). This result indicates that the optical system maintained uniform geometric magnification within approximately 2.8% across its field of view.

To quantify how the optical performance of the system translates into effective tip tracking, the execution time and robustness of the proposed algorithm were evaluated on 1920 × 1080 grayscale sequences of actuated IPMC samples. Two tracking schemes were compared: Method 1, which performs an exhaustive full-frame search for the tip, and Method 2, which employs the grid-based tip tracking algorithm described in Section 2.4.

On the reference platform, Method 1 required approximately 200 ms per frame, corresponding to an effective processing rate of about 5 frames per second, and is therefore not suitable for real-time operation. In contrast, Method 2 reduced the mean processing time to roughly 10 ms per frame when the tip was successfully detected in the first candidate region. When the prediction was less accurate and the tip was found in the second or third region, the latency increased to approximately 20 ms and 30 ms, respectively. Even in these less favorable cases, the computation remained compatible with real-time monitoring at frame rates close to the 30 fps acquisition frequency of the camera.

The reliability of the grid-based approach was assessed for motion frequencies below 2 Hz, corresponding to typical actuation conditions for IPMCs. Under these conditions, Method 2 achieved a frame-level detection accuracy of 99.4% when the search was allowed to extend to the first five candidate regions. When the search was restricted to the first three regions, the detection accuracy remained high at 98.3%. These results confirm that the prediction mechanism, combined with the adaptive expansion strategy, keeps the true tip location within a small neighborhood of grid cells for the vast majority of frames.

The experimental validation was performed up to 2 Hz, which is consistent with typical IPMC actuation under stable conditions and with the computational budget of the tracking pipeline. The proposed tip-localization runs at ~10 ms/frame in most cases and up to 20–30 ms/frame when an expanded search is required; at higher frequencies the increased inter-frame motion can reduce robustness of the prediction or local-search strategy. Extending the range is feasible via higher frame rates, larger adaptive search windows or improved motion models.

To further reduce latency, parallel execution of the detection process was investigated by splitting the region of interest into two subregions that are processed concurrently on separate cores. Each subregion corresponds to an area of 240 × 180 pixels (1920/8 × 1080/6), which contains the expected motion envelope of the IPMC tip. Parallelization led to a reduction of approximately 42% in the time required for corner detection, effectively keeping the total per-frame processing time close to the 10 ms target on low-power multicore processors such as the Raspberry Pi.

The impact of grid resolution on speed and robustness was also examined by testing three different configurations for Method 2. In the 6 × 8 configuration (48 regions of 240 × 180 pixels), the tip was typically detected within the first 2–3 regions and the average processing time was about 10 ms per frame. This configuration achieved a good balance between search locality and robustness. A denser 10 × 20 grid (200 regions of 96 × 108 pixels) was then evaluated. The smaller cell size reduced the computation per region when the prediction was correct, leading to slightly lower mean processing times in ideal conditions. However, when the prediction occasionally failed, the number of regions that had to be examined increased substantially and the overall execution time could rise sharply. In practice, this configuration was advantageous only when the prediction accuracy exceeded approximately 97%.

For completeness, a coarser 3 × 4 grid configuration (12 regions of 640 × 270 pixels) was also tested. In this case, each region covered a larger area, increasing the computational cost per region but reducing the total number of regions that needed to be checked. This scheme resulted in slower average processing times compared to the 6 × 8 grid but exhibited more stable behavior when the prediction deviated from the true trajectory of the tip. Overall, the 6 × 8 grid was found to offer the most favorable compromise between accuracy, robustness and consistent execution time, whereas the 10 × 20 grid provided the lowest latency only under highly stable motion conditions.

Combining the spatial resolution of 0.536 ± 0.001 μm/pixel on the horizontal axis and 0.518 ± 0.001 μm/pixel on the vertical axis with the 10 ms mean processing time of the grid-based method, the system can reliably resolve sub-micrometer displacements of the IPMC tip at video rates. The results demonstrate that the proposed microscopy and tracking framework enables high-precision, real-time monitoring of IPMC actuation in aqueous environments, without the need for more complex and costly laser-based measurement systems.

Finally, to validate the protection and monitoring circuitry described in Section 2.3, a series of fault-injection experiments was performed. In total, 100 fault events (25 per fault type: supply overvoltage, supply undervoltage, open circuit and short circuit) were deliberately introduced into the system. All injected faults were detected correctly, yielding a 100% detection rate with no missed events. During 3 h of continuous nominal operation, no spurious triggers were observed, confirming that the protection circuitry reliably discriminates between true fault conditions and normal IPMC actuation and can be safely used for long-term automated measurements.

## 4. Conclusions

This work demonstrated an integrated system for high-resolution displacement measurement of ionic polymer–metal composite (IPMC) actuators in aqueous environments by combining custom optical microscopy with a grid-based tip tracking algorithm and dedicated protection electronics. The experimental setup achieved a spatial sampling of 0.536 ± 0.001 μm/pixel on the horizontal axis and 0.518 ± 0.001 μm/pixel on the vertical axis, enabling reliable tracking of very small specimen motions. Lens distortion was found to be limited, with a maximum edge deviation of +0.015 μm/pixel (≈+2.8% relative to the center), indicating that geometric magnification is consistently within approximately 2.8% across the field of view. The grid-based tip tracking algorithm sustained a mean processing time of 10 ms per 1920 × 1080 frame with ≈99% frame-level detection accuracy for actuation frequencies below 2 Hz, thereby supporting real-time monitoring of sub-micrometer IPMC displacements at video rates. In addition, the integrated fault-detection circuitry successfully identified all 100 injected overvoltage, undervoltage, open-circuit and short-circuit events, with no spurious triggers during 3 h of nominal operation, ensuring safe long-term use of the system.

Beyond accuracy and speed, the proposed approach offers practical advantages in cost, implementation complexity and experimental observability. It relies on commercially available optical components and a USB camera, avoids the stringent alignment requirements of laser-based techniques and the speckle-pattern constraints of Digital Image Correlation, and provides direct visual access to both the actuator and its surrounding medium. These attributes make the system suitable for real-time micro-sensing tasks and broadly applicable to IPMC characterization, microscale biomimetic platforms and the development of advanced displacement sensors for biomedical and industrial applications. Future work will extend the methodology to higher actuation frequencies, three-dimensional trajectory reconstruction and the simultaneous tracking of multiple actuators, further broadening the range of micro-scale systems that can benefit from the proposed framework.

The present study focuses on system integration, calibration, and robustness of the tip-tracking algorithm. We did not include an independent displacement reference, so the absolute measurement error in micrometers is not fully characterized. As a result, our claims regarding spatial resolution are based on the calibrated pixel size and qualitative tracking performance.

## Figures and Tables

**Table 1:** Comparison of representative ionic polymer actuator displacement measurement approaches in terms of resolution/accuracy, indicative cost level and key advantages/limitations.

Ref	Measurement Approach	Accuracy/Spatial Resolution	Cost	Pros	Cons
This work	Optical microscopy + predictive tip tracking	0.536 × 0.518 μm/pixel	low	sub-μm sampling; non-contact; underwater-capable; low cost	limited FOV
[10]	Camera-based vision tracking	reported accuracy ~0.2 mm (640 × 480), ~0.4 mm (320 × 240)	low	simple; non-contact; underwater-feasible	mm-level accuracy; limited by camera resolution/frame rate
[14]	Laser triangulation displacement sensor	repeatability 0.5 μm; working distance 150 ± 40 mm	high	high precision; high sampling; non-contact	high cost; alignment & surface-reflectivity sensitivity
[33]	DIC + SEM micrographs	sub-pixel DIC matching (>0.01 px); SEM images 1280 × 1040 px (quasi-static)	high	full-field deformation/strain; high spatial detail	SEM vacuum (not aqueous); quasi-static only

**Table 2:** Imaging parameters and calculated spatial resolution of the microscopy system.

Parameter	Value
Slide divisions	10 µm (0.01 mm)
Image size	1920 × 1080
Field of View (FOV)	1.03 mm (W) × 0.56 mm (H)
Spatial Resolution (Width)	0.536 ± 0.001 µm/pixel
Spatial Resolution (Height)	0.518 ± 0.001 µm/pixel

## Data Availability

The data presented in this study are available on request from the corresponding author.

## Abbreviations

The following abbreviations are used in this manuscript:

IPMC	Ionic Polymer–Metal Composite
LDV	Laser Doppler Vibrometry
EMI	Electromagnetic interference
DIC	Digital Image Correlation
ADC	Analog-to-Digital Converter
DAC	Digital-to-Analog Converter
GPIO	General-Purpose Input/Output
